# 19p13.2 Microdeletion including *NFIX* associated with overgrowth and intellectual disability suggestive of Malan syndrome

**DOI:** 10.1186/s13039-016-0282-4

**Published:** 2016-09-22

**Authors:** Hai-Yun Dong, Hui Zeng, Yi-Qiao Hu, Li Xie, Jian Wang, Xiu-Ying Wang, Yi-Feng Yang, Zhi-Ping Tan

**Affiliations:** 1 Intensive Care Unit, Central South University, Changsha, Hunan Province 410011 China; 2 Clinical Center for Gene Diagnosis and Therapy of State Key Laboratory of Medical Genetics, The Second Xiangya Hospital of Central South University, 139# Renmin Road, Changsha, Hunan 410011 China; 3 Department of Cardiothoracic Surgery, Central South University, Changsha, Hunan Province 410011 China; 4 The State Key Laboratory of Medical Genetics, School of Life Sciences, Central South University, Changsha, Hunan Province 410011 China; 5 Department of Pediatrics, the Second Xiangya Hospital, Central South University, Changsha, Hunan Province 410011 China

**Keywords:** Deletion 19p13.2-13.13, Deletion 5q35.2, NSD1, SNP-array, Epigenetic diseases, Microarrays-based candidate gene strategy

## Abstract

**Background:**

Overgrowth syndromes represent clinically and genetically heterogeneous conditions characterized by a wide spectrum of malformations, tall stature, intellectual disability and/or macrocephaly.

**Results:**

In a cohort of four clinically characterized patients with overgrowth syndrome without known causative gene mutation, we performed an Illumina SNP-array analysis to identify the pathogenic copy number variations. We identified two rare copy number variations harboring overgrowth syndrome related genes. Patient 1 was Malan syndrome with a 1.4 Mb 19p13.2-13.13 microdeletion including *NFIX*, and Patient 2 was identified as Sotos syndrome with a 1.6 Mb 5q35.2 microdeletion encompassing *NSD1*.

**Conclusions:**

We identified two patients associated with Manlan syndrome and Sotos syndrome respectively. We also discuss the use of the microarrays-based candidate gene strategy in Mendelian disease-gene identification.

**Electronic supplementary material:**

The online version of this article (doi:10.1186/s13039-016-0282-4) contains supplementary material, which is available to authorized users.

## Background

Overgrowth with multiple birth defects is useful phenotypic sign in diagnostic categorization of congenital diseases. Overgrowth syndromes (OGSs) are a group of heterogeneous disorders including Sotos syndrome (SOTOS1, OMIM#117550), Beckwith-Wiedemann syndrome (BWS; OMIM#130650), Simpson-Golabi-Behmel syndrome (SGBS1, OMIM#312870), Proteus syndrome (PS, OMIM#176920), Bannayan-Riley-Ruvalcaba syndrome (BRRS, OMIM#153480) and Weaver Syndrome (WVS, OMIM #277590) [1]. Recently the molecular causes of several OGSs have been revealed [2]. Despite these advances, the underlying genetic mechanisms for 20–40 % of overgrowth individuals remain unknown [2].

Malan syndrome, also named as Sotos-like syndrome or Sotos syndrome 2 (SOTOS2, OMIM#614753), is a recently introduced clinical condition characterized by tall stature, intellectual disability and/or macrocephaly [2–4]. This syndrome has been associated with the *NFIX* gene on chromosomal locus 19p13.2. *NFIX* encodes the nuclear factor I X (CCAAT-binding transcription factor) protein [5]. It has been suggested that either a nonsense mutation in the *NFIX* gene or microdeletion encompassing *NFIX* underlies this syndrome [2, 6].

As part of a larger study on the identification of pathogenic copy number variations in children with birth defects, four overgrowth children carrying multiple birth defects with undetermined genetic reasons were enrolled in this study. High-resolution SNP-array analysis (Illumina, San Diego, CA, USA) was performed to analyze the genomes of these patients. Here, we present two patients with overgrowth syndromes, Patient 1 was diagnosed as Malan syndrome with a 1.4 Mb deletion in chromosomal region 19p13.2-13.13, and Patient 2 was identified as Sotos syndrome with a 1.6 Mb 5q35.2 microdeletion.

## Clinical report

### Patient 1

**Fig. 1:**
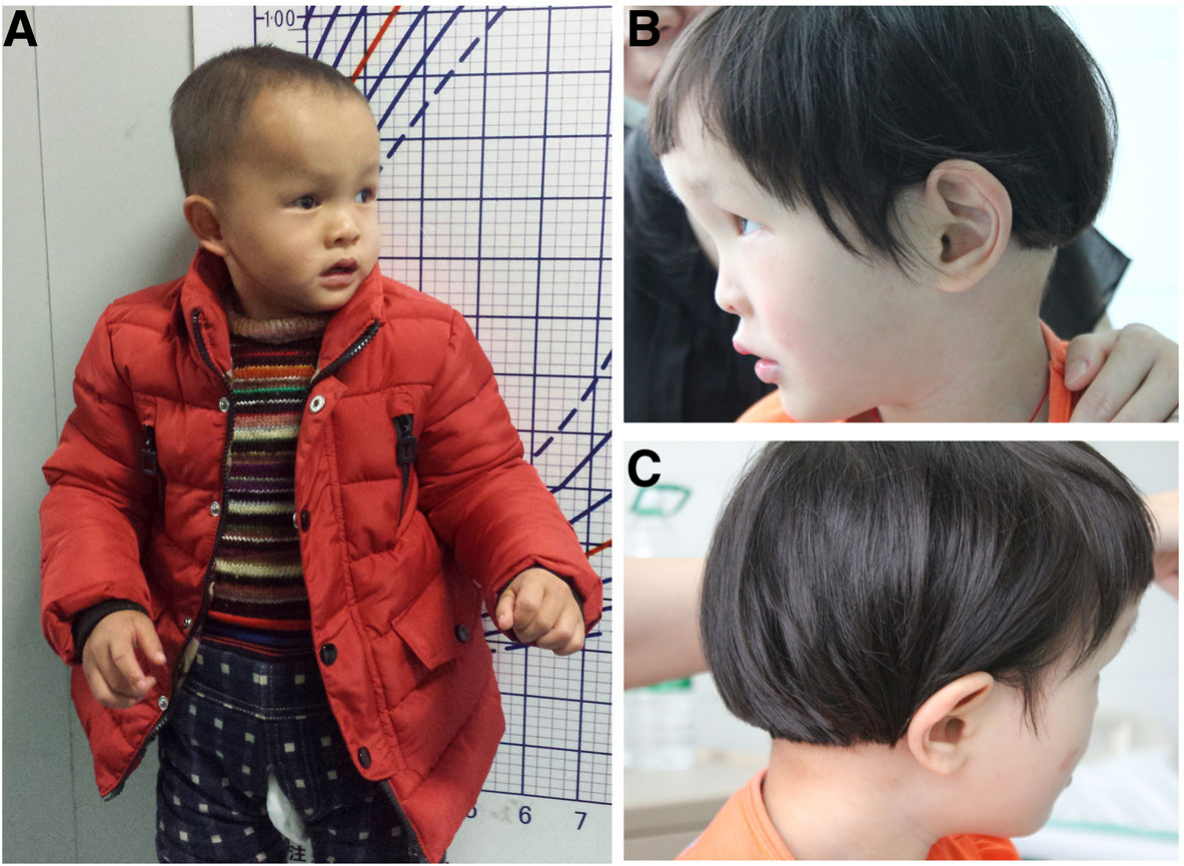
Clinical photographs of the probands. **a**: Patient 1 with Malan syndrome shows frontal bossing, high forehead, deep-set eyes, sparse eyebrows, a flat nasal bridge, low-set ears, high-arched palate, and bilateral strabismus. **b**, **c**: lateral view of the Patient 2 with Sotos syndrome. Frontal bossing, high forehead, deep-set eyes, heterochromia and congenital microphthalmia were observed

This male patient was two-year and seven-month old at the time of genetic evaluation. He was the second child of a healthy unrelated couple. Family history of birth defects or genetic disorders was denied. He was born at 39 weeks of gestation by normal delivery. His birth weight was 4000 g (75-90th centile) with a birth length of 51 cm (50th centile) and a head circumference of 37 cm (75–90th centile). Due to respiratory failure, the patient was treated with intubation and artificial respiration and admitted to the neonatal intensive care unit (NICU). At six months, he attended to the Second Xiangya Hospital because of development delay. At this time, he weighed 8000 g (75–90th centile) with a length of 72 cm (50th centile) and a head circumference of 43 cm (75–90th centile). Mildly reduced volume of the white matter, and the thin corpus callosum were revealed by brain MRI. Electroencephalogram (EEG) showed no definite abnormality. At present, his length is 79 cm (50th centile), weight 12 kg (50th centile) and OFC 48.6 cm (75–90th centile), suggesting that his head is relatively large compared with his stature. He showed frontal bossing, high forehead, deep-set eyes, sparse eyebrows, a flat nasal bridge, low-set ears, high-arched palate, bilateral strabismus and pectus excavatum (Fig. 1). He could not sit up and walk by himself. Also he could not speak any meaningful words. His psychomotor development was evaluated by the GESELL Developmental Diagnosis Scale (GDDS) and the developmental quotient (DQ) was determined to be 19. Neurological examination revealed generalized hypotonia and reduced deep tendon reflexes. Peripheral nerve conduction velocities showed no abnormalities. Conventional G-banding analyses showed no abnormalities.

### Patient 2

The patient was referred to the genetic service at the age of three years with intellectual disability, macrocephaly and congenital heart defect (patent ductus arteriosus, PDA). This girl was born to non-consanguineous, healthy parents. At her birth, her mother and father were both at 36 years old. She was born by cesarean at 39 weeks plus 4 days with a birth weight 3,780 g (70–90th centile); birth length of 52 cm (90th centile) and a head circumstance of 36.8 cm. Frontal bossing, high forehead, deep-set eyes and congenital heart disease were observed. Eye anomalies, heterochromia and congenital microphthalmia were also observed. Conventional G-banding analyses showed no abnormalities.

### Patient 3

Patient 3 attended to our hospital due to congenital heart disease. Family history was absent. He was six-year old and had several distinct phenotypes, i.e. ASD (atrial septal defect), tall stature and behavior problems (self-hugging and frequent temper outbursts).

### Patient 4

This patient was a five-year old boy. He was the second child of unrelated parents without obvious family history of inherited disease. Congenital heart defect (PDA), frontal bossing and high forehead, macrocephaly, big toe and also behavior problems (impulsiveness, frequent temper tantrums and outbursts) were observed.

## Methods

The Review Board of the Second Xiangya Hospital of the Central South University approved this study. All subjects consented to this study.

Chromosome analysis was performed on peripheral blood of the patient and the parents by conventional G-banded techniques (550-band resolution). 5 ml peripheral blood was collected for each individual. All samples were subjected to lymphocyte culture according to standard cytogenetic protocol.

The genomic DNA was prepared from peripheral blood of the patient and the parents. Genomic DNA was prepared using a DNeasy Blood & Tissue Kit (Qiagen, Valencia, CA) on the QIAcube automated DNA extraction robot (Qiagen, Hiden, Germany).

Genomic DNA samples of the patient and parents were adjusted to a final concentration of 50 ng/μl. The HumanOmni1-Quad Chip (Illumina Inc., San Diego, USA) and the Illumina BeadScan genotyping system (Beadstation Scanner) were employed to obtain the signal intensities of SNP probes [7, 8]. Human Omni1-Quad Beadchip contains over 1.1 million loci across the human genome. The GenomeStudio V2011 software was used to analyze the genotypes (human genome build 37/Hg19 for analysis) and evaluate the experimental quality. The call rates of the samples are greater than 99.5 %.

## Results

**Fig. 2:**
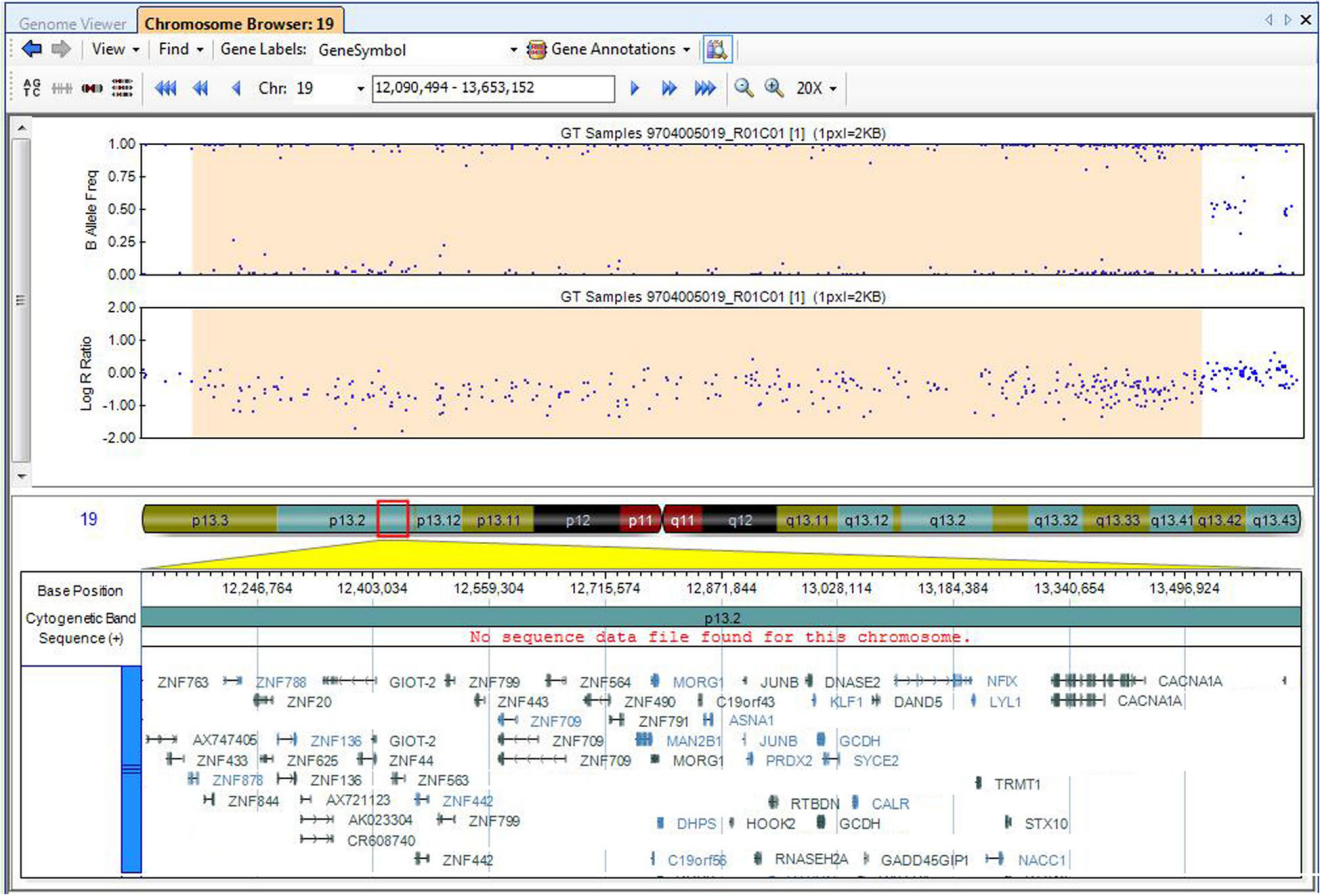
Human Omni1-Quad SNP-array result of the 19p13.2-p13.13 in Patient 1 with Malan syndrome. SNP-based array shows a *de novo* 1.4 Mb deletion (chr19: 12,157,839-13,518,462/hg19). Log R ratio and B allele frequencies are showed in upper panel; the lower panel shows genes in the deleted region

Conventional G-banding analyses showed no abnormalities in four children with overgrowth syndromes. To identify the patients' genetic lesions, we employed SNP-array system (HumanOmni1-Quad Chip) to analyze the possibility of pathogenic copy number variations (pCNVs). A *de novo* 1.4 Mb deletion ranging from 19p13.2 to 19p13.13 (chr19: 12,157,839-13,518,462/hg19) was identified in Patient 1 with unique clinical phenotypes i.e. macrocephaly, overgrowth and intellectual disability. This chromosome region is gene-rich and contains several OMIM genes *NFIX*, *CACNA1A* and *CAL*R (Fig. 2). Consistent with previous reports that deletions of 19p13.2 are associated with overgrowth and other abnormalities, this patient got a clinically diagnosis as Malan syndrome (19p13.2 microdeletion syndrome). In addition, a *de novo* 1.6 Mb deletion on chromosomal locus 5q35.2 encompassing *NSD1* (chr5:175,548,190-177,145,695/hg19) was revealed in Patient 2 with Sotos syndrome. SNP-array analysis showed no pathogenic deletions or duplications in Patient 3 and Patient 4, hence these two patients remain undiagnosed.

## Discussion

Overgrowth syndromes are a group of conditions with heterogeneity and share several cardinal clinical features, i.e., overgrowth, intellectual disability and/or macrocephaly. The complexity of phenotypic features makes the differential diagnoses of overgrowth syndromes difficult. Recent advances in genetic studies have facilitated the molecular delineation of overgrowth syndromes. As a result, several overgrowth syndromes have been genetically elucidated in last decade. In this study, we report two patients with distinct overgrowth syndromes, Malan syndrome (19p13.2 deletion of *NFIX*) and Sotos Syndrome (5q35.2 deletion of *NSD1*).

In 2010, Malan et al. combined chromosomal microarrays and candidate gene approaches and successfully discovered nonsense mutations or microdeletion of *NFIX* in patients with Sotos-like overgrowth syndrome [6]. Mutations in *NFIX* had been linked to a known clinical condition, Marshall-Smith syndrome (MSS, OMIM#602535)[9]. It is suggested that different types of *NFIX* variants underlie distinct clinical phenotypes [6]. Specifically, frameshift and splice mutations in *NFIX* resulted in Marshall-Smith syndrome (MSS) while *NFIX* deletions and nonsense mutations cause a Sotos-like overgrowth syndrome. This phenomenon that distinct mutations result in strikingly different phenotypes has also been observed in other syndromes i.e. *PTEN* mutations cause Cowden syndrome (CS) and Bannayan-Riley-Ruvalcaba syndrome (BRRS) and Proteus syndrome (PS)[10], while *FBN1* underlies Marfan syndrome, Weill-Marchesani syndrome (WMS) and acromicric dysplasia.[11, 12]. Subsequently, several reports confirmed *NFIX* mutations in patients with Sotos syndrome-like features resembling the patients previously reported by Malan et al. [2, 3, 13, 14]. In 2015, Klaassens et al., reported six additional patients with point mutation or deletion of *NFIX* [2]. Literature review of patients with 19p13.2-19p13.13 deletions showed similar clinical features [15–20]. Finally this medical condition was collectively grouped as Malan syndrome (19p13.2 deletion syndrome).

Common and prominent features are shared by different types of overgrowth syndromes, which imply that these syndromes might share similar molecular mechanisms. Since the two overgrowth disease-related genes, *NFIX* and *NSD1*, are both transcription factor, we suspect that they might be involved in similar molecular processes. The transcription factor nuclear factor I X plays a pivotal role during the development of brain and skeleton [5]. The Nfix(-/-) mice have retarded growth and most would die after postnatal day 21 (P21) in addition to malformations in brain ventricles and the corpus callosum [5]. *NSD1* encodes a protein containing a SET domain, four plant homeodomain (PHD) finger regions and a proline-rich region. The SET Domain of NSD1 protein has intrinsic histone methyltransferase activity [21]. Based on these observations, Sotos syndrome and Sotos-like overgrowth syndrome have been suggested to be epigenetic diseases caused by loss-of-function defects of epigenetic readers/writers that modify the H3K36 histone mark [22]. Consistent with this idea, mutations in the DNA methyltransferase gene *DNMT3A*, *SETD2* and *EZH2* have also been shown to cause overgrowth syndromes [22–24]. Most recent *NFIX* function studies focus on its role in the brain malformations, whether *NFIX* is involved in epigenetic regulation remains to be elucidated [25, 26].

The discovery of *NFIX* as a causal gene for Malan syndrome also provides a success example for the microarrays-based candidate gene strategy in the identification of Mendelian disease-genes. Various mapping approaches rely upon karyotyping, linkage analysis, homozygosity mapping, GWAS and the recently introduced chromosomal microarrays (copy number variation analysis) and WES/WGS (whole exome/genome sequencing) [27]. An overview of literatures suggests that 13 disease-causing genes have been successfully identified by microarrays-based approaches including array CGH and SNP-array (Table 1). The disease gene number is relatively small but shows great growing potential. More importantly, with the accumulated copy number variation database, microarrays-based candidate gene strategy would be more productive in combination with exome sequencing data (Next Generation Sequencing, NGS).

**Table 1:** Mendelian causative genes identified by the microarrays-based candidate gene strategy

Gene	Locus	Size (kb)	Syndrome/phenotype	Year	Reference
*RAI1*	17p11.2	4000	Smith–Magenis syndrome	2003	Nat Genet.2003; 33:466-8.
*CHD7*	8q12	2300	CHARGE syndrome	2004	Nat Genet. 2004; 36:955-7.
*EHMT1*	9q34	1200	9q34 deletion syndrome	2005	J Med Genet. 2005; 42:299-306.
*MECP2*	Xq28	400–800	Intellectual Disability	2005	Am J Hum Genet. 2005; 77:442-53.
*MAPT*	17q21.31	600	17q21.31 microdeletion syndrome	2006	Nat Genet. 2006; 38:999-1001.
*CHRNA7*	15q13.3	1500	15q13.3 microdeletion syndrome	2008	Nat Genet. 2008; 40:322-8.
*MBD5*	2q23.1	200–4000	2q23.1 microdeletion syndrome	2010	Eur J Hum Genet. 2010; 18: 436-441.
*NFIX*	19p13.2	100–200	Sotos-like syndrome	2010	Am J Hum Genet. 2010; 87:189-98.
*SHANK3*	22q13.3	100–9000	Phelan-McDermid Syndrome	2011	Nature. 2011; 472: 437-42.
*KANSL1*	17q21.31	68	17q21.31 microdeletion syndrome	2012	Nat Genet. 2012; 44:639-41.
*ARIDIB*	6q25	2500	Intellectual Disability	2012	Am J Hum Genet. 2012; 90:565-72
*KCTD13*	16p11.2	600	Intellectual Disability	2012	Nature. 2012; 485:363-7.
*TGFB2*	1q41	6500	Thoracic aortic aneurysm	2012	Nat Genet. 2012; 44:922-7.
*KDM6A*	Xp11.3	45–816	Kabuki syndrome	2012	Am J Hum Genet. 2012; 90:119-24.

Both of our patients have eye problems. Patient 1 has strabismus which is typical findings in patients with Malan syndrome. Patinet 2 has congenital microphthalmia and heterochromia. The latter two symptoms are not commonly reported in Sotos syndrome. In addition, Patient 1 with Malan syndrome shows no heart defects, while Patient 2 has congenital heart disease that mostly associated with Sotos syndrome. Whether the cardiac defect of our Sotos syndrome patient is related to other deleted genes in this 5q35.2 deletion remains to be resolved.

## Conclusion

In conclusion, we report two patients with overgrowth syndrome. Patient 1 is diagnosed as Malan syndrome with a 1.4 Mb 19p13.2-13.13 microdeletion including *NFIX*, while Patient 2 has Sotos syndrome with a 1.6 Mb 5q35.2 microdeletion encompassing *NSD1*. Our study contributes to the diagnosis and treatment of these diseases.

## Additional files

Additional file 1: Table S1. SNP-array data of Patient 1. (XLSX 13 kb) Additional file 2: Table S2. SNP-array data of Patient 2. (XLSX 26 kb) Additional file 3: Table S3. SNP-array data of Patient 3. (XLSX 30 kb) Additional file 4: Table S4. SNP-array data of Patient 4. (XLSX 29 kb)

## Abbreviations

ASD: Atrial septal defect
*CACNA1A*: Calcium voltage-gated channel subunit alpha1 A
*CALR*: Calreticulin
CGH: Comparative genomic hybridization
CHD: Congenital heart disease
*DNMT3A*: DNA methyltransferase 3 alpha
DQ: Developmental quotient
EEG: Electroencephalogram
*EZH2*: Enhancer of zeste 2 polycomb repressive complex 2 subunit
*FBN1*: Fibrillin 1
GWAS: Genome-wide association study
*NFIX*: Nuclear factor I X
*NSD1*: Nuclear receptor binding SET domain protein 1
OFC: Occipital frontal circumference
OGS: Overgrowth syndromes
OMIM: Online Mendelian Inheritance in Man
pCNVs: Pathogenic copy number variations
PDA: Patent ductus arteriosus
PHD: Plant homeodomain
*PTEN*: Phosphatase and tensin homolog
*SETD2*: SET domain containing 2
SNP: Single nucleotide polymorphism
WES/WGS: Whole exome/genome sequencing
